# End-of-life decisions guiding the palliative care of cancer patients visiting emergency department in South Western Finland: a retrospective cohort study

**DOI:** 10.1186/s12904-018-0383-4

**Published:** 2018-12-17

**Authors:** Outi M. Hirvonen, Jenni E. Alalahti, Kari J. Syrjänen, Sirkku M. Jyrkkiö

**Affiliations:** 1 Department of Oncology and Radiotherapy, Turku University Hospital, PO Box 52, FI-20521 Turku, Finland; 2 Department of Clinical Oncology, University of Turku, Turku, Finland; 3 Department of Clinical Research, Biohit Oyj, Helsinki, Finland

**Keywords:** End-of-life project, Palliative care, Cancer, Emergency department, Advance care planning, Palliative outpatient clinic

## Abstract

**Background:**

Until recently, palliative care (PC) resources in Finland have been sparse. To meet the increasing need for PC an end-of-life (EOL) care project has been ongoing in South Western Finland since 2012, and in 2015, a weekday palliative outpatient clinic was established in Turku University Hospital (TUH). The aim of this study was to explore the effect of the project and the PC clinic on the management practices of EOL cancer patients attending the Emergency Department (ED) of TUH from 2013 to 2016.

**Methods:**

The medical records of all cancer patients (ICD-10 codes C00–97) admitted to the ED of TUH between August 1–December 31, in 2013 and 2016, were analyzed: *n* = 529, *n* = 432 respectively (2013 and 2016). The analysis focused on those patients in EOL care; *n* = 77, *n* = 63, respectively. The late palliative patients were defined by PC decision, thus termination of life-prolonging cancer-specific treatments. The EOL patients were in the imminently dying phase of their illness. The site of referral after an ED visit was also verified together with the documentation on advance care plans (ACP), and the impact of palliative outpatient visits.

**Results:**

In 2016, the number of late palliative and EOL patients admitted to the ED has shown a tendency to decrease. The quality of the documentation for treatment goals, do-not-resuscitate (DNR) orders, living wills and connections to primary care providers has improved since 2013. Prior visits to palliative outpatient clinic correlated well with the more comprehensive ACP information: i) DNR order (*p* = 0.0001); ii) connection to primary care (*p* = 0.003); iii) documented ICD-10 code Z51.5 (*p* = 0.0001).

**Conclusions:**

Even modest investments in resources for PC can induce an objective change in the allocation of health care resources, and improve the ACP for the cancer patients at their EOL. A visit to a palliative outpatient clinic may offer one approach for improving the quality and completion of ACP documentation.

## Background

Cancer patients often need hospital care during the last months of their life [1]. An increased number of ED visits is characteristic of patients not receiving sufficient palliative care, and an indicator of poor quality of care for patients with terminal cancer, or cancer at the end of their life [2, 3]. On the contrary, in-home PC patients are less likely to visit the ED or be admitted to the hospital than those receiving standard care [4], and community-based PC is associated with reduced ED visits [5].

For patients dying of cancer, a visit to the ED is often distressing and exhausting. While some patients do have urgent medical problems that demand ED attention, many ED visits are potentially avoidable. The percentages of potentially avoidable ED visits have varied from 20% to 51,5% in the studies focusing on this issue [6–8]. There is only sparse data regarding the emergency medical needs of cancer patients at the EOL [3, 9].

Studies suggest that many ED physicians feel underqualified to treat PC patients [3]. Clear documentation of the goal of the treatment in the patient’s medical records is a vital guide for decision-making processes in the ED [10]. Prior advance care planning, including the connection to the primary health care, a DNR order and a living will, as well as the documentation of ICD-10 code Z51.5 (palliative care) would help clinicians working in busy ED’s. It would especially help them to provide appropriate treatment for patients nearing the end of their lives without undue delay.

The clinical practice guideline of the American Society of Clinical Oncology (ASCO), update January 2017 [11], recommends that every patient with advanced cancer should receive dedicated PC services early in the course of the disease, and concurrently with active treatment. However, until recently, the level of PC has only been at the preliminary health system integration in Finland [12], thus the awareness of PC on the part of health care professionals as well as policy makers has arisen but the systematic PC services are in the phase of an active development. Also, the number of palliative units in the country remains limited [13].

The aim of this study was to evaluate the impact of the end-of-life project and the palliative outpatient clinic on the number, quality and outcome of ED visits towards the end of life for patients with cancer.

## Methods

### Study design

This was a retrospective registry-based cohort study before-and-after the EOL project and establishment of weekday palliative outpatient clinic.

### Study setting

The study was performed in Turku University Hospital (TUH), which is the main hospital in the Hospital District of Southwest Finland and one of the five university hospitals in Finland. It is responsible for the cancer care for 0.47 million residents, and comprising an area of 10,900 km^2^. The hospital district is formed by communities, which duty is to provide both primary and secondary health care to all residents in the county. The tax-financed Finnish health care system provides cancer care for all residents of the district with minimal cost to the patients. The ED of the hospital is available 24 h every day of the week to all citizens in the area. The number of patients visiting the ED has increased from approximately 57,000 in 2013 to 67,000 in 2016. In the study, secondary care refers to the wards in the university hospital, whereas primary care refers to the wards in PC units of the regional communities. Primary care services are located near patients’ homes and are less expensive for the communities and patients.

The project for the development of EOL care was started in the hospital region in 2012, and is still going on. It consists of a plan and description of how EOL care should be organized in the hospital district. The guideline for the project states that good care should be organized close to the patients’ home with the support of a home care team and a responsible physician; these recommendations follow the ASCO palliative care guidelines [11, 14]. A proactive ACP should be offered to every patient including proper symptom management, the possibility of admission to a primary care ward in the community without a visit to the hospital ED at any time of the day, and the restrictions placed on the care provided (e.g. a DNR order) accompanied by the patients’ own wishes (e.g. living will with a surrogate). As part of the development project, regular regional education and an EOL network for both hospital and community based primary care physicians and nurses were established. Thus, lectures of symptom management, psychosocial support, organization of PC as well as other issues of PC were arranged along with group meetings where experiences, good practices of delivering PC and contact info were shared.

In TUH, a palliative outpatient clinic was established in 2005, however, because of the scarce resources a more systematic palliative intervention only became available in 2015. In 2013, the palliative outpatient clinic only consisted of a part-time palliative specialist and a nurse, however, in 2015 these positions were supplemented by a full-time doctor sub-specializing in PC, and a further full-time nurse. As a result, in 2013 there were 273 patients visiting the clinic, and by 2016 the number of patients had risen to 732.

As well as the investment in a PC outpatient clinic, educational training for the personnel working in primary care in the hospital district was also emphasized; investments into specialized palliative home care teams were also carried out by the municipalities in South Western Finland. In addition, new palliative ward services for EOL patients were established in the region. These wards allow EOL patients to be accepted onto the ward 24 h every day of the week, based on a phone call from the patient or a family member. Moreover, the palliative outpatient unit of the hospital systematically started to prepare proactive care plans for the patients, using the ICD-10 code of Z51.5 (PC) and discuss with patients DNR orders and living wills.

### Cohort selection

All cancer patients admitted to the ED of the TUH from August 1 to December 31, 2013 were reviewed. During the later period of the project, all cancer patients admitted to the ED from August 1 to December 31, 2016 were reviewed accordingly and a comparison of the results was performed. The time points represent the beginning of weekday palliative outpatient clinic and education for the hospital and community based staff in 2013, and the evaluation of the effects of these efforts evaluated at 2016. Patients were identified from the ED discharge records by ICD-10 codes C00-97. Only the first ED visit for each patient during the 5-month period was included.

### Variables

The number of all patients visiting the ED of TUH in the study period in 2013 and 2016 were searched for, as well as the number of cancer patients. The recorded patient characteristics of cancer patients included age, gender, and cancer diagnosis. If the patient had more than one cancer diagnosis, only the diagnosis patient was been treated at the moment was included in the analysis.

Patients were classified into four categories according to the goal of patient’s cancer treatment: I) curative, II) early palliative, III) late palliative or IV) EOL (by authors OH and JA). For these definitions, all the medical records of the patients were used. Early palliative treatment included ongoing or planned disease-modifying or stabilizing chemo- and radiotherapy as well as biological cancer therapies. The late palliative group included patients not receiving chemo- or biological therapies, i.e. treatment aiming at life prolongation. However, short courses of radiotherapy e.g. to relieve pain was allowed in this group. The EOL patients were terminal, as documented in the medical records. Thus these patients were in the imminently dying phase of their illness, and in patient records of these patients the term “EOL decision” for recorded decision to start comfort only EOL care was searched for.

The age, gender and cancer diagnosis were analyzed from all cancer patients of the study. In addition, survival of the patients was evaluated from the hospital records 6 months after their ED visit. Hospital records are updated regularly according to Statistics of Finland in order to secure the accurate information on survival of the patients treated at our hospital (www.stat.fi).

Patients classified in categories III or IV (late palliative or EOL) were analyzed further by retrieval of information regarding the indication for admission and the place of discharge from the ED. The indications for admission included inability to cope at home, definitive surgical problem, pain, dyspnea, infection and other reason. The place of discharge contained home, secondary care ward, Turku city hospital, primary care ward, other place (e.g. hospice). In addition, it was verified whether the patient had deceased during the treatment period and if the goal of treatment was documented clearly in the medical records of the patient (by authors OH and JA). The connection to the primary care as well as the documentation of the DNR order and the living will were recorded. The connection to the primary care included either community based PC services or a primary care physician. From the data, we also determined whether the ICD-10 code Z51.5 was registered, and whether the patient had regularly visited the palliative outpatient clinic prior to the ED admission.

### Data sources

In order to obtain all the information needed for the study, all the medical records of the hospital were used. In TUH all documentation is systematically collected in electronic medical records. The present study is based on the hospital registry data, and, since the data has already been collected for clinical purposes, no patient interventions were performed; therefore, the legislation does not mandate any ethics committee approval. The study was evaluated, registered and approved by the institutional board of TUH.

### Statistical analysis

Data management followed the principles of the Declaration of Helsinki [15]. All statistical analyses were performed using the IBM SPSS Statistics for Windows, version 24.0.0.2. (IBM, New York, USA) software. Frequency tables were analyzed using the χ^2^-test, with the likelihood ratio (LR) or Fisher’s exact test for categorical variables. All statistical tests were two-sided and considered statistically significant at *p*-values < 0.05.

## Results

**Fig. 1:**
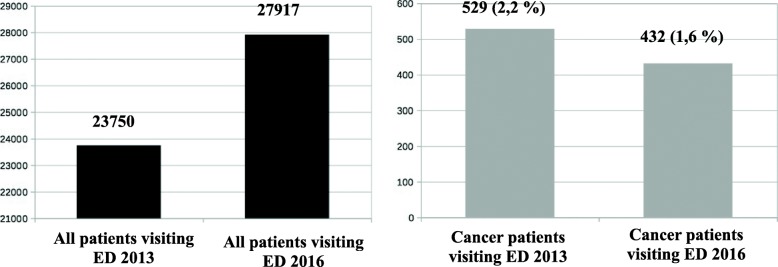
The number of all patients visiting ED of TUH along with number of cancer patients visiting ED from August 1 to December 31, both in 2013 and 2016. The percentages of cancer patients from all patients are also shown

A total of 529 and 432 individuals with a cancer diagnosis were admitted to the ED during the 5-month study period in 2013 and 2016, respectively (Fig. 1). Accordingly, these patients represent 2,2 and 1,6% of all the patients visiting the ED. A total of 47 and 62 patients (9 and 14% of the cancer patients, respectively) received their cancer diagnosis during the ED visit or at the following examination. These patients were excluded from further analyses, and consequently 482 (2013) and 370 (2016) patients were left in the study. The characteristics of the patients are shown in Table 1.

**Table 1:** The characteristics of all cancer patients visiting the ED of the TUH from August 1 to December 31, in 2013 and 2016, respectively

Characteristics	8–12/2013	8–12/2016
Number of all patients (pts) visiting the ED	529	432
Pts whose cancer was diagnosed following the ED visit (excluded)	47	62
Mean age	68	68
Min./Max.	16–98	20–94
Gender:		
Female	216 (45%)	175 (48%)
Male	266 (55%)	195 (52%)
Number of pts whose cancer had been diagnosed earlier	482	370
Diagnosis groups		
Gastrointestinal cancer	87	67
Breast cancer	65	56
Lung cancer	61	47
Prostate cancer	65	32
Leukemia	31	22
Lymphoma	23	17
Pancreas cancer	17	21
Gynecological cancer	26	15
Bladder cancer	35	21
Myeloma	16	14
Kidney cancer	9	13
Brain cancer	18	10
Head & neck cancer	10	4
Other cancers	19	31
Total	482	370

The overall survival rate of cancer patients who received treatment with a curative intention was good (16 and 30% of the cancer patients, respectively, 6 months OS 87 and 90%). The early palliative group of patients was the largest group to visit the ED (65 and 45%, accordingly), and nearly half of these patients died within 6 months (OS 61 and 51%). The number of patients in the late palliative group was small (14 and 17%), and most of them died within 6 months (OS 13 and 26%). The smallest group consisted of patients in the EOL group. The number of patients in the EOL group decreased from 2013 to 2016 (1.7, 0.5%). All these patients died within 6 months. The goal of the treatment was not defined for a small number of patients (2.9 and 8.1%) because their cancer had been so recently diagnosed that there had not been enough time to plan the treatment. The characteristics of the late palliative and EOL cancer patients is shown in Table 2.

**Table 2:** The characteristics of late palliative and EOL cancer patients visiting the ED of TUH from August 1 to December 31, in 2013 and 2016, respectively

Characteristics	8–12/2013	8–12/2016	*p*-value
Number of late palliative and EOL pts visiting the ED^a^	77	63	*p* = 0.30
Late palliative pts	67	61	
EOL pts	10	2	
Mean age	73	74	NS
Gender			*p* = 0.004
Female	31	41	
Male	46	22	
Diagnosis groups^b^			*p* = 0.014
Gastrointestinal cancer	36	15	
Lung cancer	11	7	
Prostate cancer	10	8	
Breast cancer	5	3	
Other cancers	22	30	
Total	77	63	
Indication to the ED visit			*p* = 0.965
Inability to cope at home	18	15	
Definitive surgical problem	14	15	
Pain	11	8	
Dyspnea	11	7	
Infection	9	6	
Other	14	12	
Total	77	63	

^a^ The late palliative group included patients not receiving chemo- or biological therapies. However, short courses of radiotherapy e.g. to relieve the pain was allowed in this group. The EOL group consisted of imminently dying patients

^b^ The four largest groups of diagnosis are presented, and the other cancers included in the group “other cancers”

**Fig. 2:**
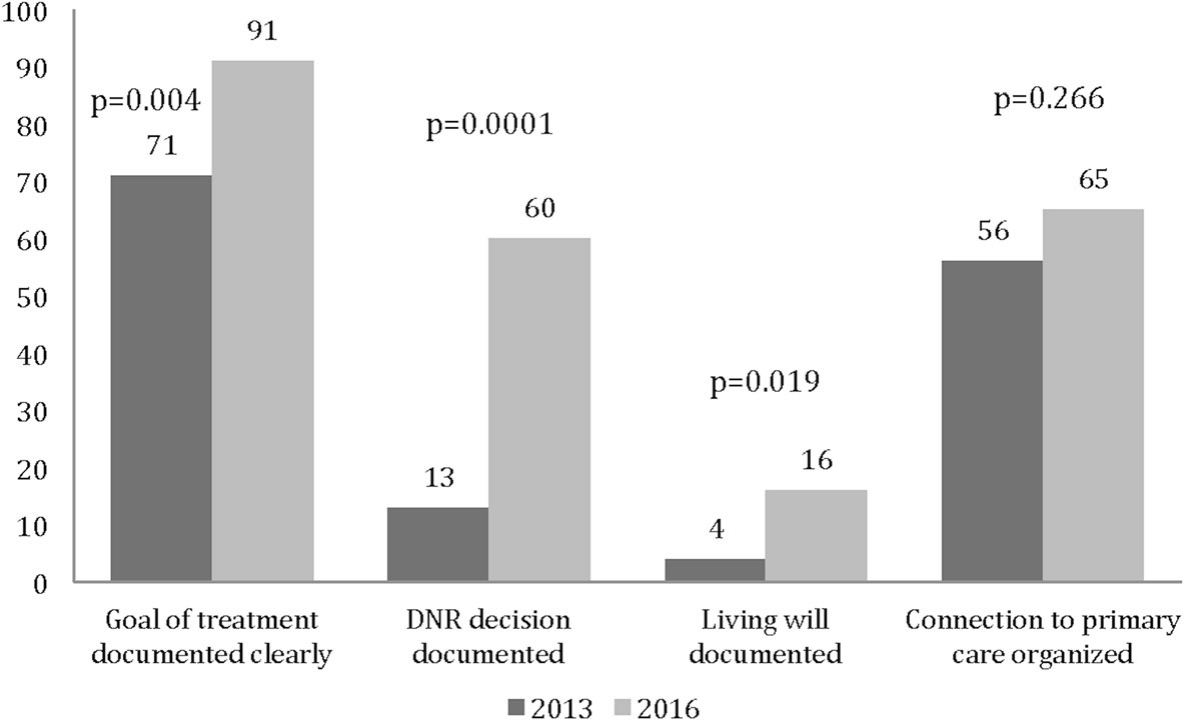
The percentages of the documentation of the integral issues of the ACP for the late palliative and EOL cancer patients of TUH, Finland in 2013 and 2016

The place of discharge from the ED tended to change over the research period from a secondary care ward to a primary care ward. Thus the place of discharge was secondary care ward in 46% of the patients in 2013 and in 37% of the patients in 2016. The respective numbers of the patients to be discharged to primary care ward were 12 and 25%. Both in 2013 and 2016, a quarter of the patients of the patients were discharged home. The percentages of the patients who died during the hospital period decreased in the study period (39 and 29%, respectively). The DNR orders were significantly better documented in 2016 than in 2013 (Fig. 2). Although living wills were hardly ever documented, the number of living wills documented had, however, increased during the observation period.

**Fig. 3:**
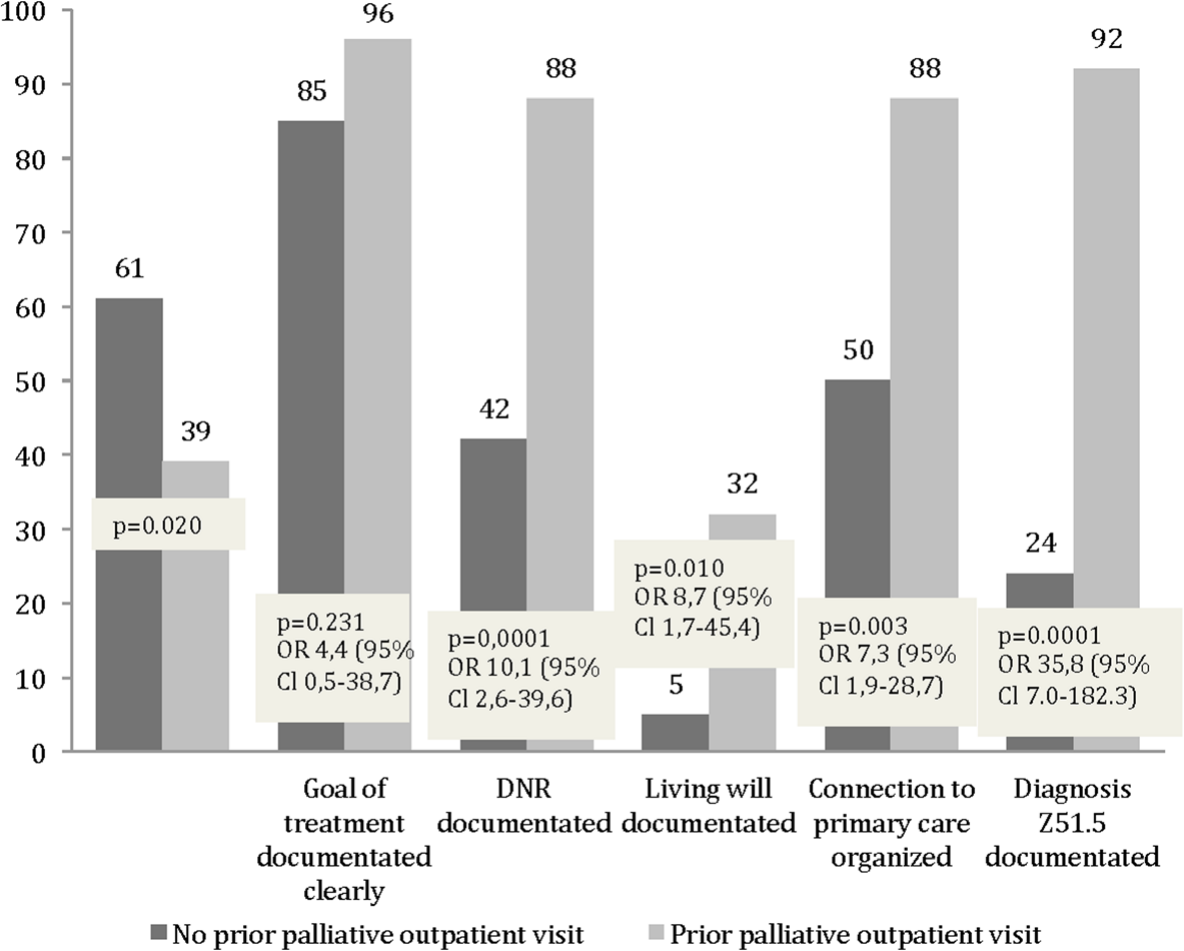
The percentages of palliative outpatient visits and the influence of this intervention on the documentation of the ACP for the late palliative and EOL cancer patients in 2016

Even though there were more resources as regards the palliative outpatient clinic in 2016, still less than half of the patients had visited the palliative outpatient clinic in 2016 (39%, Fig. 3). The DNR documentation increased significantly for those patients who had visited the palliative outpatient clinic (88% vs 42%, *p* = 0.0001) and a connection to primary care providers was more often organized (88% vs 50%, *p* = 0.003). In addition, the ICD-10 code Z51.5 was clearly documented for the patients who had visited the palliative outpatient clinic.

## Discussion

After the EOL project there was a difference observed and an association found between PC outpatient visits and documentation of DNR orders as well as completion of ACPs. Additionally, the place of discharge from the ED tended to have changed from secondary to primary care. The prior visits of late palliative and EOL patients to the outpatient palliative clinic of TUH significantly changed the documentation of the DNR orders, living wills, and much more importantly, significantly facilitated the connection with the primary care services near the patient’s home. The admissions of these patients to the ED tended to decrease, thus reducing the resources needed. Education and network activities are important in order to increase the level of know-how as regards PC for all professionals at different levels in the health care system, however, in order to enhance the PC of patients, more structured palliative interventions are of profound importance.

One quarter of the patients visited the ED because of their inability to cope at home, which implies that the patient had no clear medical pathway for direct admission. In these cases, the patient usually had several symptoms, e.g. fatigue, an impaired general condition, and malaise. Deterioration of the general condition of patients with advanced cancer is probably due to the progress of the disease and, as such, is not an indication of the need for ED admission; therefore, it should be more systematically treated by primary care providers. From the health care system’s point of view, patients dying of cancer make important and well-founded demands on the total resources of the hospital sector, and any reduction in the utilization of hospital wards is also beneficial from a socioeconomic perspective [16].

A substantial proportion of the late palliative and EOL care patients visiting the ED had no connection with their local primary care (Fig. 2). However, the number of patients with this connection did increase over time, in particular if the patient had visited the palliative outpatient clinic. Despite the earlier studies indicating that PC services decrease the likelihood of ED visits and hospital admittance [2, 3, 12, 13], consultation with supportive care does not necessarily decrease the overall use of ED visits [17, 18]. Our data shows similar results i.e., that palliative intervention needs active effort and the resources for 24 h every day of the week- interventions of PC for patients living at home at the EOL are of the utmost importance in order to avoid unnecessary secondary care admissions. Unfortunately, the resources for these interventions in the district remain insufficient.

Documentation of DNRs and living wills were more comprehensive for patients with prior visits to the palliative outpatient clinic. This is in line with previous studies reporting that PC consultation increased the completion of advance directives [19, 20]. Furthermore, only a minority of patients with a prognosis of less than 1 year, reported having had discussions about EOL care with their oncology clinicians. Nevertheless, the mortality of these patients was high and majority had no ACP [21]. This emphasizes the importance of a distinct PC intervention being made by a palliative outpatient clinic in addition to the usual oncologic visits. Despite the significant increase in the resources of the palliative outpatient clinic of the hospital during the study period, in 2016, the outpatient clinic was only visited by less than half of late palliative and EOL patients. According to the medical records, some of these patients had their first appointment scheduled in the near future and others did not, because their oncology clinician had not made a referral. Improvement is clearly needed as regards the earlier timing of referrals.

A direct comparison between studies is difficult regarding the proportion of patients reported as having made a DNR order or a living will since the patient populations in the studies differ significantly. In recent studies of patients with advanced cancer [22–25], the percentages of DNR orders have differed from 18 to 41%, and living wills from 28 to 53%, respectively. Over the research period the percentages of DNR orders in our study improved considerably. In 2016, the percentages were higher compared to other studies, and the palliative outpatient visits had further enhanced these orders. However, the policy for the documentation of living wills needs improvement. Considering that our patients were at a late palliative or EOL stage of the disease, both of these documents should have been in order.

Our results are in accordance with prior studies, and show that interdisciplinary PC interventions result in higher numbers of completed advance directives and overall supportive care referrals [26]. The study by Clark and co-workers also stated that although physicians have an important role in facilitating ACP discussions, involvement of other staff, e.g. nurses and social workers is associated with a greater likelihood of the completion of ACP documentation [27]. Similarly in our clinic nurses and social workers have an important role in communicating these topics with patients.

Nearly half of the patients in the early palliative group of patients had died within 6 months of their ED visit. This highlights the ASCO clinical practice guideline recommendation that every patient with advanced cancer should receive dedicated PC services, early in the course of the disease, and concurrent with active treatment [11]. Presently, at TUH it is rare for a patient to visit the palliative outpatient clinic before life prolonging treatment has been completed, i.e. in the early palliative phase of the disease. In order to change this practice, referrals by oncologists to the palliative outpatient unit should be facilitated.

The limitations of this study include those that are inherent in retrospective studies. This was a small-scale single-center study, although quite comprehensive since it included all ED visits in the catchment area of one University Hospital. We cannot exclude the possibility that some patients were diagnosed in the ED with a non-oncological diagnosis such as an infection or atrial fibrillation. Our data were collected from the medical records of ED visits and prior medical records. However, we did not determine whether some of these patients underwent EOL discussions later on, e.g. during their hospitalization. In addition, we were unable to assess whether some of the patients were incapacitated and therefore unable to make EOL decisions for any reason. Even in such cases, however, the discussion could have been conducted with a surrogate.

## Conclusion

Even fairly modest investments in the resources for PC and education for professionals treating EOL patients can make a veritable change in how health care resources are used, and improve the ACP of the patients. A visit to a palliative outpatient clinic may offer one approach for improving the quality and completion of ACP documentation. Consequently, services of the patients at the EOL should be addressed outside ED.

## Abbreviations

ACP: Advance care plan
DNR: Do-not-resuscitate
ED: Emergency Department
EOL: End-of-life
PC: Palliative care
TUH: Turku University Hospital
